# The role of the autonomic nervous system in polycystic ovary syndrome

**DOI:** 10.3389/fendo.2023.1295061

**Published:** 2024-01-19

**Authors:** Yue Yu, Tong Chen, Zheng Zheng, Fan Jia, Yan Liao, Yuehan Ren, Xinmin Liu, Ying Liu

**Affiliations:** ^1^ Guang’anmen Hospital, China Academy of Chinese Medical Sciences, Beijing, China; ^2^ Wuxi Hospital Affiliated to Nanjing University of Chinese Medicine, Wuxi, China

**Keywords:** polycystic ovary syndrome, autonomic nervous system, sympathetic nervous systems, parasympathetic nervous systems, norepinephrine, heart rate variability

## Abstract

This article reviewed the relationship between the autonomic nervous system and the development of polycystic ovary syndrome (PCOS). PCOS is the most common reproductive endocrine disorder among women of reproductive age. Its primary characteristics include persistent anovulation, hyperandrogenism, and polycystic ovarian morphology, often accompanied by disturbances in glucose and lipid metabolism. The body’s functions are regulated by the autonomic nervous system, which consists mainly of the sympathetic and parasympathetic nervous systems. The autonomic nervous system helps maintain homeostasis in the body. Research indicates that ovarian function in mammals is under autonomic neural control. The ovaries receive central nervous system information through the ovarian plexus nerves and the superior ovarian nerves. Neurotransmitters mediate neural function, with acetylcholine and norepinephrine being the predominant autonomic neurotransmitters. They influence the secretion of ovarian steroids and follicular development. In animal experiments, estrogen, androgens, and stress-induced rat models have been used to explore the relationship between PCOS and the autonomic nervous system. Results have shown that the activation of the autonomic nervous system contributes to the development of PCOS in rat. In clinical practice, assessments of autonomic nervous system function in PCOS patients have been gradually employed. These assessments include heart rate variability testing, measurement of muscle sympathetic nerve activity, skin sympathetic response testing, and post-exercise heart rate recovery evaluation. PCOS patients exhibit autonomic nervous system dysfunction, characterized by increased sympathetic nervous system activity and decreased vagal nerve activity. Abnormal metabolic indicators in PCOS women can also impact autonomic nervous system activity. Clinical studies have shown that various effective methods for managing PCOS regulate patients’ autonomic nervous system activity during the treatment process. This suggests that improving autonomic nervous system activity may be an effective approach in treating PCOS.

## 1 Introduction

Polycystic Ovary Syndrome (PCOS) stands as a prevalent multisystem disorder, characterized by disruptions in reproductive, endocrine, and metabolic functions. Due to the heterogeneity of clinical manifestations of PCOS, the diagnostic criteria for PCOS have always been a hotly debated topic. In 1990, the National Institutes of Health (NIH) preliminarily outlined the criteria for diagnosing PCOS, with clinical/biochemical hyperandrogenism and chronic anovulation being considered key for the diagnosis. The diagnosis of PCOS is established when a patient presents two of the above clinical criteria while ruling out other potential causes of hyperandrogenism, such as congenital adrenal hyperplasia, Cushing’s syndrome, and androgen-secreting tumors. The NIH criteria for PCOS did not mention polycystic ovary (PCO), although there was controversy, it was widely used before the Rotterdam criteria appeared in 2003 (1). In 2003, the European Society of Human Reproduction and Embryology and the American Society for Reproductive Medicine formulated the Rotterdam diagnostic criteria for PCOS, proposing that at least two of the following three criteria were mandatory: oligo-anovulation, clinical/biochemical hyperandrogenism, and PCO appearance on ultrasonography. This included the ultrasonographic features of polycystic ovarian morphology (PCOM) in the diagnostic criteria (2). The introduction of the Rotterdam criteria significantly increased the number of patients diagnosed with PCOS. In 2006, the Androgen Excess and PCOS Society (AE-PCOS) proposed that in addition to meeting the criteria of oligo-anovulation or PCO, clinical or biochemical hyperandrogenism should be essential for the diagnosis of PCOS (3). The 2018 International Evidence-based Guideline updated the diagnostic criteria for PCOS based on the 2003 Rotterdam Consensus criteria, stating that anti-Müllerian hormone (AMH) can be used instead of ultrasound in the diagnosis of PCOS (4).

Influenced by diagnostic standards, geographical factors, and ethnic/racial factors, the prevalence of PCOS in women varies. Azziz reported a cumulative prevalence of 6.6% in women of reproductive age in Alabama, USA, with no significant statistical difference between black and white women, using the NIH 1990 criteria (5). A prospective study on the prevalence of PCOS in Spain, using the NIH 1990 criteria, reported a prevalence of 6.5% (6). From October 2007 to September 2011, an epidemiological study of 15,924 Han women of childbearing age in 10 provinces and cities of China, referring to the Rotterdam criteria, found a PCOS prevalence of 5.6% (7). According to the NIH 1990 criteria, the prevalence of PCOS in indigenous women aged 15-44 in Darwin and its surrounding areas in the Northern Territory of Australia was 15.3% (8). In South America, a study reported the prevalence of PCOS was 8.5% in women seeking primary health care in Salvador, Brazil, according to the Rotterdam criteria (9). Another study showed that the prevalence of PCOS among the Caucasian population in Ankara, Turkey, as reported according to the NIH, Rotterdam, and AE-PCOS Society criteria, was 6.1%, 19.9%, and 15.3%, respectively (10).

Despite its widespread occurrence and substantial impact on women’s health, the precise pathogenesis of PCOS remains incompletely understood, and effective treatment options are currently lacking. Women with PCOS are at increased risk of Obstructive sleep apnea (OSA) (11), hypertension (12), depression (13), obesity (14), type 2 diabetes (15), and the pathogenesis of these diseases is highly associated with the imbalance of the autonomic nervous system (ANS) (16–20).

The ANS encompasses the sympathetic nervous system, parasympathetic nervous system, and enteric nervous system. The enteric nervous system is intrinsic to the gastrointestinal tract and collaborates with the parasympathetic and sympathetic systems to regulate digestion and absorption (21). While the function of the whole body is mainly regulated by the sympathetic nervous system and the parasympathetic nervous system. Nerve function is mediated by neurotransmitters, acetylcholine (ACh) and norepinephrine (NE) being the dominant autonomic neurotransmitters. In the parasympathetic system, both pre- and postganglionic neurons release ACh, with presynaptic ACh primarily acting on nicotinic receptors within autonomic ganglia and postsynaptic ACh primarily affecting muscarinic receptors in effector organs. In the sympathetic system, preganglionic neurons also release ACh, while postganglionic neurons release NE, which serves as the primary sympathetic neurotransmitter acting on α and β adrenergic receptors in target organs (22). The sympathetic and parasympathetic systems interact antagonistically, cooperatively, or independently to provide neural control over nearly all body tissues except skeletal muscle. In response to external and internal perturbations, the ANS regulates processes such as vascular tone, body temperature, heart rate, and glandular secretion to maintain homeostasis. In a healthy state, the autonomic nervous tone maintains an active equilibrium (23).

Apart from its role in maintaining the physiological activities of normal tissues and organs, the ANS is also implicated in the pathogenesis of various diseases. Imbalance of ANS, that is, abnormal increase or decrease of sympathetic or parasympathetic nervous system tension, may be related to the occurrence and development of PCOS. This review article aims to consolidate the latest developments in research pertaining to the relationship between the ANS and PCOS. It explores the role of these neural systems in the onset and progression of PCOS and discusses potential strategies for rectifying ANS balance as a means of treating PCOS. This research provides novel insights into disease prevention and management.

## 2 Autonomic nervous participate in the regulation of reproductive function

Mammalian ovarian function is under the control of the ANS, which operates in conjunction with the hypothalamic-pituitary-ovarian axis (HPOA), collectively influencing the secretion of ovarian steroid hormones and the process of ovulation (24, 25). In rat, the ovaries receive sympathetic innervation through the ovarian plexus nerves (OPN) and superior ovarian nerve (SON). The left OPN originates from the lumbar ganglion of the sympathetic trunk (LGST), while the right OPN arises from both LGST and the superior mesenteric ganglion (SMG). After entering the ovary, these OPN nerves travel along the ovarian artery, innervating both the ovarian medulla and cortex regions (26). They may play a role in corpus luteum development and maturation (27). SON originates from the suprarenal ganglion (SG) and communicates with the ovaries through SG, Celiac ganglion (CG), and SMG. SON projects into the ovarian suspensory ligament, running parallel to its long axis, and enters the ovary through the ovarian hilum, distributing within the ovarian stroma and around the follicles, it is distributed in the ovarian stroma and around the follicle, and innervates blood vessels, interstitial glands and ovarian endometrial cells (28). SON contributes to ovarian function by influencing follicular development and steroidogenesis.Approximately 90% of NE in the ovaries is derived from the sympathetic nervous system. NE acts on β2-adrenergic receptors present in ovarian theca cells and granulosa cells, stimulating androgen production, enhancing follicle recruitment, and thereby increasing the likelihood of cystic follicle formation, which can trigger PCOS (29–31). Studies have shown that compared to non-PCOS women, PCOS patients have an increased density of adrenergic nerve fibers, significantly elevated levels of NE and dopamine (DA) in follicular fluid. DA is a precursor to NE and can be converted to NE after absorption by oocyte cells (32). NE may lead to elevated local oxidative stress levels in the ovaries, potentially impairing follicular development (33).

Nerve Growth Factor (NGF) is a marker of sympathetic neural activity. As a neurotrophic factor regulating the adrenergic neurons of the ovary, NGF provides nutritional support for the development of peripheral sympathetic nerve fibers (34). The follicular membrane is a critical area for the distribution and regulation of sympathetic neurons in the ovary, where NGF and its two types of receptors can be synthesized in follicular membrane cells (35). NGF is involved in the development and maintenance of follicles. However, excessive NGF can damage the bidirectional communication between oocytes and cumulus cells, significantly inhibiting the meiosis and maturation of oocytes (36). Dissen reported that NGF levels in the follicular fluid and granulosa cell culture medium are elevated in PCOS patients compared to non-PCOS populations (37). Studies have shown that transgenic mice overexpressing NGF in the follicular membrane (17NF mice) exhibit a higher density of tyrosine hydroxylase-positive nerve fibers in the ovaries, indicating an overdominance of sympathetic nerves. These mice display pathological characteristics of PCOS, such as arrested development of antral follicles, hyperandrogenemia, polycystic ovarian changes, accompanied by increased granulosa cell apoptosis and persistent elevation of serum luteinizing hormone (LH) levels. They also exhibit reproductive dysfunction along with metabolic abnormalities such as hyperinsulinemia, increased body fat, and insulin resistance (IR). Ovarian blockade of NGF can reduce the formation of cystic follicles induced by EV in PCOS rats, restoring their estrous cyclicity and ovulation (38). Furthermore, Manti using 17NF mice found that ovarian overexpression of NGF leads to placental dysfunction, impaired embryonic development, and offspring exhibiting increased sympathetic output until adulthood, with irregular estrous cycles, abnormal morphology and function of adipose tissue, and impaired glucose metabolism, reflecting reproductive-metabolic complications characteristic of PCOS (39). Kisspeptin is a class of neuropeptides widely distributed in the human body, participating in the regulation of the dynamic balance of female reproductive functions. Research indicates that Kisspeptin can stimulate the release of gonadotropin-releasing hormone (GnRH), playing a significant role in the hypothalamic-pituitary-gonadal axis (40). In addition to regulating the release of gonadotropins, Kisspeptin may also directly regulate follicular development through paracrine or autocrine mechanisms (41). A meta-analysis based on 699 patients and 583 controls pointed out that, compared to non-PCOS patients, patients with PCOS have higher serum levels of Kisspeptin, kisspeptin levels were independently correlated with PCOS (42). Elevated serum levels of Kisspeptin in PCOS patients may lead to an overactive hypothalamic-pituitary-gonadal axis (43). In the ovaries and corpus luteum, Kisspeptin levels are regulated by the sympathetic nervous system. The use of the β-adrenergic agonist isoproterenol increases Kisspeptin expression levels, while the β-adrenergic antagonist propranolol can reverse this increase (44, 45).

The vagus nerve is an essential component of the parasympathetic nervous system, with its ascending fibers terminating in four nuclei in the dorsal medulla: the dorsal motor nucleus of the vagus (DMV), solitary nucleus (NTS), trigeminal spinal nucleus, and ambiguous nucleus. There is no direct link between the gonads and the vagus nucleus in the central nervous system (46). The vagus nerve reaches the ovaries via peripheral nerves, with the left vagus nerve passing through the esophagus, bifurcating before inserting into the stomach, and entering the right celiac ganglion.The right vagus nerve runs parallel to the esophagus and is formed by the small ganglion into the right vagus plexus, which makes a connection with the celiac plexus and joins the RCG. The vagus nerve establishes connections with SON and OPN through the anterior vertebral ganglia. SON and OPN convey information from both vagus nerves to the ovaries, allowing indirect reception of central nervous system information by the ovaries (47). Because the vagus nerve innervates the gastrointestinal system, Through the synaptic connection of neurons in the prevertebral ganglion of the celiac plexus, information can be transmitted between the central nervous system, the stomach and the ovary, suggesting vagal innervation may play a role in the metabolism of the ovary (48). The vagus nerve is cholinergic fiber, and ACh is its primary neurotransmitter. ACh is synthesized from choline and acetyl coenzyme A (AcCoA) through choline acetyltransferase (ChAT). Choline is transported to the presynaptic nerve terminal via Na-dependent choline transporters. Adult rat and human ovaries contain ChAT enzymes, indicating that, in addition to exogenous ACh arriving via the vagus nerve, the ovaries themselves can synthesize ACh. Research has shown that granulosa cells (GC) of developing follicles and corpus luteum cells can produce ACh, and ACh secretion is influenced by gonadotropins, providing nutrition for follicular development and ovulation through muscarinic receptors (49–51). A decrease in ACh levels can interfere with the growth of antral follicles and reduce reproductive capacity (52). Acetylcholinesterase (AChE) is also present in the ovaries and can hydrolyze ACh into choline and acetate (53). Studies have suggested that a splice variant of AChE, AChE-R, can induce necrotic apoptosis in granulosa cells, leading to follicular atresia and corpus luteum dissolution. Disrupting AChE enzyme activity may be a novel pathway affecting ovarian function (54). Administration of the AChE inhibitor huperzine A (HupA) to the ovaries of rats increased ovarian ACh levels and secondary follicle numbers after 4 weeks, significantly increasing corpus luteum numbers, indicating improved ovulation rates (55).

## 3 Autonomic nerves and PCOS in animal models

### 3.1 Estradiol valerate induced rat model

The use of animal models enriches the pathophysiological research of PCOS and serves as a crucial tool in exploring the mechanisms and treatments for PCOS. In studies investigating the relationship between PCOS and the ANS in animal models, estrogen-based modeling is the most commonly employed method. This involves a single intramuscular injection of long-acting estrogen, estradiol valerate (EV), at doses of 2-4 mg per rat to induce PCOS models (56). Exposure to EV during the critical developmental window causes irreversible changes in ovarian function, typically occurring between 14 and 24 days of age in Sprague-Dawley (SD) rats (57). EV induction results in disrupted estrous cycles, anovulation, and significant polycystic changes in the ovaries. Hormone levels such as follicle-stimulating hormone (FSH), LH, and testosterone (T) are altered. Because T can be rapidly converted into estradiol (E2) within the ovaries, the T levels in EV-induced rats may vary, showing reductions (58, 59), elevations (60), or similarities to control groups (61). The fundamental principle of EV induction is that high estrogen levels increase the pituitary gland’s sensitivity to GnRH, leading to elevated LH levels and FSH suppression, thereby establishing a typical endocrine environment characteristic of PCOS and inducing non-obese PCOS animal models (62).

Changes in autonomic nervous activity occur in EV-induced rats with PCOS. Heart rate variability testing in PCOS rats reveals reduced cardiac vagal tone and increased sympathetic activity compared to normal rats (63). Studies have shown that, 60 days after EV injection, the ovaries of rats exhibit polycystic changes. At 30 days, there is a significant increase in the ovarian neurotransmitter NE released by sympathetic nerves. This is accompanied by selective downregulation of β-adrenergic receptors in the ovarian follicular membrane, indicating that activation of sympathetic neurons that innervate the ovaries precedes the onset of ovarian polycystic changes (64). Modulating the ANS that innervates the ovaries can impact the development of PCOS in rats. Neosaxitoxin is an algal toxin that binds to the outer pore of voltage-gated sodium channels, specifically blocking neuronal voltage-dependent Na^+^ channels. This interaction produces an effective and reversible blockade of nerve conduction. Studies have demonstrated that Neosaxitoxin has minimal hemodynamic effects (65, 66). Ovarian cells are excitable, and endocrine-type voltage-dependent Na^+^ channels, sensitive to Neosaxitoxin, are present in the ovaries (67). Animal experiments indicate that local administration of Neosaxitoxin to the ovaries via a micro-osmotic pump can reduce Na^+^ levels in a short period. After 28 days of Neosaxitoxin application, a decrease in NE levels was also observed. This suggests that Neosaxitoxin chronically inhibits sympathetic nerve activity induced by EV in rats. Such intervention increases the number of corpora lutea, reduces ovarian follicular cysts, and lowers plasma T levels, restoring normal estrous cycles in rats (68).

The drug guanethidine selectively acts on postganglionic adrenergic nerve endings, antagonizing NE release. Administering guanethidine to EV-induced rats prior to successful modeling prevents ovulatory disturbances and hyperandrogenism (69). Beyond pharmacological interventions, neural regulation is also an intervention approach. Given the communication between peripheral and central nervous systems, one study removed the right ovarian tissue of EV-induced PCOS rats, eliminating the assumed influence of the right ovary on the hypothalamus. Subsequently, they removed the left SON and found that, compared to sham-operated rats, rats with severed nerves exhibited reduced numbers of cystic follicles and restored ovulation (70). Kilohertz-frequency alternating current (KHFAC) modulation is an emerging bioelectronic application that allows reversible regulation of nerve activity compared to nerve excision. In a study, researchers surgically removed the right ovary of rats and implanted electronic devices into the left SON to suppress nerve firing rates. After 2-3 weeks of KHFAC modulation, irregular or absent estrous cycles in PCOS rats were reversed, corpus luteum numbers increased, and ovarian NE concentrations decreased (71).

Vagal nerve transmission is also involved in the development of PCOS. In 24-day-old PCOS rats induced by EV, unilateral or bilateral vagotomy improved estrous cycles, restored ovulation, reduced ovarian androgen production, and decreased ovarian NE. However, there was no change in NE concentration in the celiac superior mesenteric ganglia complex (CSMG), which serves as a hub for the ovaries to receive information from the central system. This suggests that the vagus nerve can regulate ovarian function by directly affecting ovarian NE activity, independent of gonadotropin influences (72, 73). It has also been pointed out that the vagus nerve plays an asymmetric role in regulating the ovarian NE concentration, and the left vagotomy leads to a decrease in NE concentration, while the right vagotomy does not change NE concentration (74).

The ANS may influence PCOS rats through various mechanisms. Animal experiments have shown that low-frequency electroacupuncture (EA) stimulation can increase ovarian blood flow in PCOS rats. However, severing the SON and OPN nerves eliminates the increased ovarian blood flow, indicating that ovarian blood flow response to EA is mediated by the ANS. Ovarian blood flow affects follicular development. Thus, regulating ovarian blood flow may be one of the mechanisms by which the ANS affects PCOS (75). Using splenic macrophage culture medium to stimulate intact ovaries in PCOS rats, researchers found increased release of pro-inflammatory molecules such as TNFα and NO by splenic macrophages, elevated Bax/Bcl2 ratios, increased apoptosis, and a high-inflammatory state. This state was reversed when the SON was severed. Additionally, markers of sympathetic nervous activity, such as NGF and kisspeptin, were downregulated, possibly indicating activation of the splenic sympathetic nervous system anti-inflammatory pathway. This suggests that the ANS also plays a role in regulating the immune function of inflammation in PCOS (76).

### 3.2 Androgen induced rat model

Exposure to excess androgens is a significant risk factor for PCOS. Overexposure to androgens during embryonic development can predispose individuals to PCOS, and increased intrauterine androgen levels in PCOS patients greatly increase the risk of passing PCOS genetically to their female offspring (77). Androgen exposure is also a commonly used method to induce PCOS models. Subcutaneous injection of T or dihydrotestosterone (DHT) in female rats can induce hyperandrogen PCOS models with reproductive dysfunction, and an increase in ovarian norepineergic fiber density, i.e. a change in ovarian sympathetic innervation, was observed after the development of cystic follicles in the model rats (78). The sympathetic nervous system is also involved in the metabolic disturbances induced by androgens in rats. There may be communication between sympathetic nerve cell bodies and fat cells, influencing lipid metabolism (79). Research has shown that PCOS rat models exhibit increased body weight, decreased sympathetic innervation in brown adipose tissue, and reduced thermogenic activity. EA can activate sympathetic innervation in brown adipose tissue and reduce body weight (80). Combining EA with exercise can improve the ovarian morphology of DHT-induced PCOS rats, increase healthy antral follicles, and decrease elevated phenotypic markers of sympathetic neurons, such as neuropeptide Y and NGF, in the mesenteric fat tissue. This provides support for the theory that sympathetic nervous activity is involved in the metabolic regulation of visceral fat in PCOS rats (81). Apart from lipid metabolic dysregulation, animal experiments have found that DHT-induced PCOS rats have increased mean arterial pressure. Treatment with adrenergic antagonist drugs like prazosin and propranolol or renal denervation can lower the mean arterial pressure in these rats, indicating that the activation of sympathetic and renal nerves were involved in the increase of blood pressure in PCOS rats (82).

### 3.3 Stress induced rat models

Sympathetic nervous system activity can be activated by stress responses. Cold stress can stimulate the release of glutamate in the paraventricular nucleus of the hypothalamus, and glutamate, through its N-methyl-D-aspartate receptor, further mediates the release of thyrotropin-releasing hormone. In this process, NE activity in the SON increases. Injecting glutamate receptor antagonists into the paraventricular nucleus reverses the activation of ovarian sympathetic nerves (83). Activation of the sympathetic nervous system promotes the development of PCOS. Rats subjected to stress exhibit halted follicular development, increased cystic follicles, elevated plasma T levels, irregular estrous cycles, infrequent ovulation, reduced fertility, resulting in PCOS-like phenotypes (84, 85). Research indicated that repeated cold exposure over four weeks can lead to sinusoid follicles with thickened follicular membranes in the ovaries of rats, which progress to cysts or type III follicles after eight weeks of stress exposure (86). Local administration of AChE inhibitor Huperzine-A (Hup-A) in the ovaries, which increases ovarian ACh levels, reduces ovarian cysts, increases corpus luteum numbers, and restores normal T and E2 plasma levels in stress-induced PCOS rats (87). Chronic mild unpredictable stress activates noradrenergic neurons in the locus coeruleus of the brainstem. The locus coeruleus is connected to pre-synaptic cell bodies in the ovarian sympathetic nerve pathway, leading to increased plasma and ovarian NE levels. This induces PCOS in rats similar to cold stress. The traditional Chinese medicine Xiao-yao-san intervention effectively improves abnormal follicular development, reduces granulosa cell apoptosis and autophagy in CUMS-induced PCOS rats. The treatment mechanism is believed to involve the reduction of locus coeruleus dopamine β-hydroxylase and c-FOS levels and the downregulation of NE and β2-adrenergic receptor expression in ovarian tissue (88).

## 4 Measurement of autonomic nervous system activity

Unlike in animal experiments where the role of the ANS is primarily judged through nerve blockade, in clinical practice, various non-invasive or minimally invasive instruments are often used to measure the autonomic nervous activity of patients. As the relationship between the ANS and the pathophysiological development of PCOS is continuously explored in basic research, the detection of autonomic nerve function in patients with PCOS is gradually being applied in clinical practice. Common clinical measurement methods include heart rate variability testing (HRV), muscle sympathetic nerve activity (MSNA), skin sympathetic response (SSR), and heart rate recovery (HRR) after exercise. HRV is the variation in the difference between each heartbeat cycle (the interval between the two consecutive R-waves of the electrocardiogram, R-R interval), resulting from the regulation of the sinoatrial node by the ANS. HRV measurement has become widely used for assessing ANS function and is currently one of the most commonly used non-invasive quantitative electrocardiographic evaluation method (89).

Analysis of HRV is often based on linear theoretical approaches, including time domain analysis and frequency domain analysis. Time domain analysis is a statistical measure of the discrete trend of R-R intervals of two adjacent heart beats, and the results of time domain analysis providing a comprehensive assessment of the ANS regulation of heart rate. Commonly used time domain indicators in clinical include the standard deviation of all N-N intervals (SDNN), the standard deviation of adjacent normal R-R intervals (SDANN), and the root mean square of successive differences (RMSSD). SDNN reflects overall HRV, SDANN is associated with long-term HRV, and RMSSD represents short-term HRV (90, 91).The frequency domain analysis method is obtained by calculating the power spectrum through Fourier transform, which is more sensitive and accurate, reflecting the energy change with frequency change in HRV. Among the frequency domain indicators, low frequency power (LF) is jointly regulated by the cardiac sympathetic and vagal nervous systems, primarily reflecting sympathetic nervous system activity. High frequency power (HF) is mainly influenced by cardiac parasympathetic nervous activity. The LF/HF ratio is used to assess the overall balance between the sympathetic and parasympathetic nervous systems, with higher values indicating a dominance of sympathetic activity and lower values indicating parasympathetic dominance. Ultra low frequency power (ULF) and very low frequency power (VLF) components are less frequently observed and typically require the analysis of continuous data spanning over 24 hours or more. These components represent diurnal rhythms, temperature regulation, and hormonal influences. Total power (TP) corresponds to the total energy of the four spectral bands (LF, HF, VLF, and ULF) and reflects the dominant influence of sympathetic nervous activity or overall ANS activity (92, 93). The nonlinear calculation of autonomic nervous function includes SD1 and SD2,where SD1 represents the short axis of the HRV scatter plot, indicating the width of the plot at half its length, and SD2 represents the long axis of the scatter plot, measuring the length along the 45-degree line (94). Commonly used clinical HRV parameters were shown in **Table 1**.

**Table 1:** Summary of the main heart rate variability parameters.

Time domain parameters			
Variable	Units	Description	Physiological origin
SDNN	ms	Standard deviation of all R–R intervals	The degree of heart rate variability
SDANN	ms	The standard deviation of the averages of R–R intervals during all 5-min periods	Sympathetic nervous activity
RMSSD	ms	Root mean square of the difference between adjacent R–R intervals	Parasympathetic nervous activity
NN50	beats	Number of pairs of adjacent R–R intervals difference by more than 50 ms	Parasympathetic nervous activity
pNN50	%	Percent of R–R intervals differing more than 50 ms from each other	Parasympathetic nervous activity
Frequency domain parameters			
Variable	Units	Description	Physiological origin
TP	ms^2^	The band ranges less than or equal 0.4Hz, the amplitude of normal heartbeat interval	Sympathetic or autonomic nervous activity
HF	ms^2^	The band ranges between 0.15 and 0.40Hz, the amplitude of the normal heartbeat interval in the high-frequency range	Parasympathetic nervous activity
HFnorm	nu	Normalized units of HF	Parasympathetic nervous activity
LF	ms^2^	The band ranges between 0.04 and 0.15 Hz, the amplitude of the normal heartbeat interval in the low-frequency range	Sympathetic and parasympathetic nervous activity
LFnorm	nu	Normalized units of LF	Sympathetic nervous activity
VLF	ms^2^	The band ranges between 0.003 and 0.04Hz, the amplitude of the normal heartbeat interval in the very low frequency range	Long-term regulation mechanisms, thermoregulation and hormonal mechanisms
ULF	ms^2^	The band ranges less than or equal 0.003Hz, the amplitude of the normal heartbeat interval in the ultra low frequency range	Circadian oscillations, core body temperature, metabolism and the renin-angiotensin system
LF/HF		Low frequency power-to-high frequency power ratio	Sympathetic activity or autonomic nervous balance
Non-linear indices			
Variable	Units	Description	Physiological origin
SD1		Quick and high-frequency changes in heart rate variability	Parasympathetic nervous activity
SD2		Long term changes in heart rate variability	Sympathetic nervous activity

MSNA is a measurement of the skin response of the sympathetic nervous system involving both MSNA frequency and MSNA burst incidence. MSNA frequency refers to the number of integrated bursts per minute, while MSNA burst incidence is the number of bursts per 100 heartbeats. MSNA reflects vasomotor activity and is a key regulator of cardiovascular homeostasis. It regulates blood pressure and blood flow through a cascade of neural vascular signals originating from the central nervous system. This neural-driven stimulus recruits efferent sympathetic neurons and can be used to assess sympathetic nervous system activation (95). Microneurography is the gold standard for assessing sympathetic vasoconstriction and vasodilation outflow, and can directly assess MSNA in humans at the neuronal level. The measurement method is to insert a tungsten microelectrode with a tip diameter of a few microns directly into the neuromuscular bundle through the skin, and find a site that can record the burst of neural activity and record MSNA directly. To facilitate identification, nerves chosen for recording need to be located close to the body surface, while also being sufficiently large to support and stabilize the microelectrode tip. Commonly selected nerves include the radial nerve, median nerve, ulnar nerve in the upper limbs, and the tibial nerve and fibular nerve in the lower limbs (96). SSR is one of the electrophysiological methods used to assess sympathetic nervous system function. When a nerve is stimulated, nerve impulses travel through large myelinated sensory fibers, passing through central synapses including the hypothalamus, brainstem, limbic system, and spinal intermediolateral column. Subsequently, they efference through pre-ganglionic and post-ganglionic sympathetic fibers, leading to the excitation of sweat gland activity in the skin, which can be recorded as a reflexive electrical potential in the skin, known as SSR (97). The SSR is usually stimulated by electrical stimulation of the median nerve or the posterior tibial nerve, and the recording electrode is placed in the palm of the hand or foot. The reference electrode is placed on the back of the hand or foot. The acting electrodes are placed in the palms and feet. Upper and lower limbs can be recorded simultaneously. The SSR latency reflects the conduction time of the nerve impulse causing sweating in the whole reflex arc, while the amplitude reflects the excitability of sweat glands with secretory activity and is a reliable indicator of peripheral sympathetic nerve activity (98, 99).

HRR after exercise is a commonly used indicator for evaluating cardiac ANS activity. It is defined as the rate at which heart rate decreases following maximal graded exercise testing. HRR is calculated as the difference between peak heart rate during exercise and heart rate at 1, 2, 3, 4, 5, and 7 minutes after exercise cessation (100). In current clinical studies, the test method of HRR value can be extreme dose, subextreme dose or symptom-restricted cardiopulmonary exercise test, and the postexercise position can be standing, sitting, supine, etc. During the early phase of post-exercise HRR, enhanced vagal activity plays a major role in the rapid decline of heart rate. Subsequently, increased parasympathetic activity and decreased sympathetic activity together contribute to the return of heart rate to baseline levels before exercise. Delayed HRR reflects compromised parasympathetic activity, reduced vagal tone, and relatively heightened sympathetic activity, resulting in a slower decline in heart rate (101). Additionally, delayed recovery of systolic blood pressure (SBP) following peak exercise may also have diagnostic value and may reflect excessive sympathetic nervous system activity (102). Respiratory motion is regulated by the ANS, and changes in respiratory rate can cause periodic changes in heart rate. The cardiovascular and respiratory centers regulate vagal nerve outflow through expiratory-driven reflexes, and reducing the respiratory rate may increase vagal nerve tension. Therefore, measuring respiratory cycles could also be an effective way to reflect whether autonomic nerve activity is balanced (103, 104). Devices like the respiratory inductance plethysmograph can non-invasively measure parameters such as respiratory rate, tidal volume, and minute ventilation, and have good prospects for clinical application (105).

## 5 Changes in autonomic nervous system function in PCOS patients

Various methods have been applied in clinical settings to investigate the potential abnormalities in ANS activity among PCOS patients, with HRV detection being the most commonly used method. Multiple clinical studies have shown that PCOS patients exhibit enhanced sympathetic nervous system activity and reduced parasympathetic nervous system activity compared to control groups of women with regular menstrual cycles (106–109). However, due to the heterogeneity of PCOS, which often involves metabolic abnormalities, these factors can also influence the ANS function in PCOS patients. For instance, Yildirir and colleagues found that PCOS women have lipid abnormalities, with lower serum levels of high-density lipoprotein cholesterol (HDL-C) and higher triglyceride levels and total cholesterol-to-HDL-C ratio compared to normal menstrual cycle controls (106). PCOS patients also tend to have higher body mass index (BMI) and blood pressure compared to control group women (107, 108). Studies have shown that in PCOS patients, indices representing parasympathetic nervous system activity, such as TP, HF, HFnorm, and SD1, are significantly higher in those with lower BMI (<25 kg/m^2^) (108), and there is a significant negative correlation between BMI and SDNN, LF, and HF (109). When combined with glycemic parameters, Saranya found a significant positive correlation between LF/HF and BMI, waist-hip ratio (WHR), and fasting blood sugar (110). Regression analysis has shown that IR index (HOMA-IR) and atherosclerosis index are independently related to the LF-HF ratio (111). Stroop color-word conflict tests used as mental stress tests (112) have revealed that anovulatory PCOS women exhibit impaired sympathetic nervous regulation following stress compared to women with regular ovulatory cycles (113). Mishra compared PCOS patients with age-matched healthy female subjects and found no significant differences in weight, BMI, waist circumference, hip circumference, and WHR between the two groups. The HRV index of the two groups at baseline was comparable, but the autonomic driving force of PCOS women decreased after exercise, indicating that young PCOS women with normal weight also had potential autonomic dysfunction, which was exposed after exercise (114).

HRR has been employed to assess cardiac ANS activity in PCOS patients. Tekin used a control group of women without hyperandrogenemia, menstrual cycle, and ovulation regularity. These women did not differ significantly from PCOS women in terms of age, BMI, triglycerides, T, and LH levels. All subjects underwent symptom-limited exercise tolerance testing using a modified Bruce protocol. The results indicated that PCOS patients had decreased 1-minute HRR, increased SBP during exercise peak, and delayed SBP recovery after exercise, indicative of decreased autonomic activity (115). Giallauria and colleagues demonstrated a negative correlation between HRR in PCOS women and BMI and insulin area under the curve (116). Similarly, Kaya and colleagues observed a decrease in 1-minute HRR in PCOS patients, along with elevated homocysteine levels, it suggests that PCOS patients have an increased risk of cardiovascular disease in the future. PCOS patients also had significantly higher total T levels, fasting insulin, and HOMA-IR compared to the control group. HOMA-IR and BMI were identified as independent determinants of abnormal HRR in PCOS patients. The study also indicated a negative correlation between the inflammatory marker C-reactive protein (CRP) and HRR, suggesting that IR, hyperandrogenism, autonomic dysfunction, and chronic low-grade inflammation may collectively play a role in the pathophysiology of PCOS (117).

Direct measurements using microneurography have confirmed elevated MSNA in PCOS patients, including both MSNA frequency and MSNA burst incidence (118–121). Sverrisdóttir and colleagues found a positive correlation between MSNA levels and T and cholesterol levels in PCOS (118). Lansdown and colleagues measured MSNA during isometric forearm contraction in subjects, and compared to matched control groups, PCOS patients exhibited enhanced MSNA. Functional magnetic resonance imaging of the right orbitofrontal cortex showed differential activation, which correlated with insulin sensitivity. Hyperinsulinemia in PCOS may affect sympathetic output (119). Lambert compared obese women with PCOS. The two groups had similar metabolic characteristics, including BMI, waist circumference, hip circumference, high-density lipoprotein and low-density lipoprotein, cholesterol, triglyceride, glucose, insulin sensitivity and blood pressure. Nevertheless, PCOS patients had increased MSNA and heightened sympathetic drive (120). Shorakae also found in clinical studies that a significant correlation between MSNA and PCOS status after adjusting for age and BMI, demonstrating that metabolic abnormalities in PCOS can influence ANS activity, but elevated MSNA remains independently associated with PCOS (121). Furthermore, the mean latency of SSR in PCOS patients was significantly delayed, and the mean amplitude was reduced,also indicating the presence of autonomic dysfunction in PCOS patients (122).The changes of autonomic nerve parameters in PCOS patients were shown in **Table 2**.

**Table 2:** Summary of research results on cardiovascular autonomic parameters.

Reference	PCOS/Controls number	PCOS patients age	Controls age	Effect of experimental group compared with control group
(80)	30/30	27.9 ± 6.1	31.4 ± 6.5	LFnorm↑、LF/HF↑、HF↓、HFnorm↓
(81)	35/32	29.91 ± 0.73	31.06 ± 0.68	LF↑、LF norm↑、LF/HF↑、HF norm↓
(82)	30/30	28.03 ± 5.33	27.27 ± 5.69	TP↓、HF↓、HFnorm↓、VLF↑、LFnorm↑、LF/HF↑、SD1↓ 、SD2↓
(83)	23/23	26.7 ± 4.8	25.4 ± 4.6	SDNN↓、RMSSD↓、LF↓、HF↓
(84)	31/30	23.129 ± 4.129	24.733 ± 2.935	LFnorm↑、HFnorm↓、LF/HF↑、SDNN↓、RMSSD↓、NN50↓、pNN50↓
(85)	35/32	23.1 ± 4.1	24.6 ± 2.9	TP↓、HFnorm↓、LFnorm↑、LF/HF↑、SDNN↓、RMSSD↓、NN50↓、pNN50↓
(87)	30/23	22.80 ± 5.80	22.65 ± 5.89	(post-stress)LFnorm↓、LF/HF↓
(88)	27/25	19.00-23.00	19.00-20.00	(after exercise)LF↓
(89)	26/24	25.5 ± 3.9	26.0 ± 3.8	TP↓、SDNN↓、SDANN↓、RMSSD↓、pNN50↓、HF↓、LF↓、VLF↓、HRR1↓、
(90)	75/75	21.7 ± 2.1	21.9 ± 1.8	HRR↓
(91)	68/68	24.2 ± 4.8	24.4 ± 3.9	HRR↓
(92)	20/18	29.9	27.4	MSNA↑
(93)	20/20	29.8 ± 4.8	29.7 ± 5.0	MSNA↑
(94)	19/21	31.3 ± 1.6	28.2 ± 1.6	MSNA↑
(95)	49/23	30 ± 6	29 ± 8	MSNA↑
(96)	37/33	21.56 ± 3.37	21.20 ± 1.85	SSR↓

↑, The experimental group was higher than the control group, and the difference was statistically significant; ↓: The experimental group was lower than the control group, and the difference was statistically significant.

In summary, PCOS has high heterogeneity, and different metabolic levels can affect autonomic nerve activity in PCOS patients. These women may already have autonomic nerve abnormalities before PCOS can be diagnosed, and autonomic nerve function detection has a good clinical application prospect for early diagnosis of PCOS. Women with PCOS are at increased risk for cardiovascular disease, and autonomic abnormalities can be effective predictors of future cardiovascular complications in women with PCOS. This also suggests that in terms of treatment, improving autonomic nervous function may help delay the occurrence and development of PCOS, in addition, autonomic nerve may be used as an evaluation indicator of patient treatment effect.

## 6 Potential mechanisms of autonomic nervous effect on PCOS

### 6.1 Interacting with adipokines

Adipose tissue is not only an energy storage for the body but also the largest endocrine organ. Through paracrine and autocrine pathways, it releases various adipokines targeting different organs and tissues. These adipokines regulate biological processes such as glucose and lipid metabolism, energy expenditure, inflammatory response, and immune reactions in the body (123–125). In PCOS, adipocyte dysfunction is observed (126). Studies indicate that compared to healthy women, PCOS patients have significantly lower serum concentrations of adiponectin (APN), and higher levels of leptin (LEP) and chemerin (CHEM). The diagnosis of PCOS is an independent predictor of serum levels of LEP, APN, and CHEM. The levels of LEP, APN, and CHEM can be used as independent biomarkers for the diagnosis of PCOS (127). The central nervous system receives information from adipokines and sends out metabolic balance signals through autonomic nervous circuits, suggesting the involvement of autonomic nerves in PCOS through regulating adipokines (128).

Secreted by mature white adipose tissue, APN possesses antioxidative, anti-inflammatory properties, and stimulates energy expenditure (129). As an endogenous insulin sensitizer, APN enhances glucose absorption by increasing fatty acid oxidation and reducing hepatic glucose synthesis, thereby improving insulin sensitivity. Sepilian et al. reported reduced serum APN levels in PCOS patients, showing a significant negative correlation with IR (130). APN also plays a role in ovarian steroidogenesis. Binding of APN and its receptors (AdipoR1 and AdipoR2) in the ovaries upregulates FSH levels, induces the synthesis of progesterone and E2, and inhibits LH and androgen levels in ovaries (131–133). Subcutaneous injection of APN in dehydroepiandrosterone-induced PCOS mice lowered their elevated weight and androgen levels, restoring ovulation (134). The synthesis of APN is regulated by the ANS. Animal studies show that cold exposure physiologically activates the sympathetic nervous system, reducing serum and white adipose tissue APN expression, reversible by β-adrenergic receptor antagonists (135). In PCOS patients, reduced APN concentration is an independent factor for the diminished HRR in PCOS women (136). Shorakae et al., using menstrually regular overweight or obese women as controls, found that after adjusting for age and BMI, the APN levels in PCOS women were lower than those in the control group, with significantly higher sympathetic nerve activity, suggesting that hyperactive sympathetic nerves may be a driving factor for reduced APN levels in PCOS (137).

LEP is an adipokine secreted mainly by white adipose tissue. It crosstalk with steroid-producing cells and can be transmitted to the brain as a metabolic signal, affecting the HPOA and regulating ovulation. A higher level of LEP will inhibit E2 synthesis and interfere with follicle development (126, 138). LEP is also involved in the immune inflammatory response of the body. Compared with women of normal reproductive age, the level of circulating LEP in women with PCOS is increased and positively correlated with Interferon-γ level. In cell experiments, interferon-gamma treatment increases the apoptosis of human granular cells (139). A meta-analysis involving 991 women with PCOS and 898 healthy control women showed that LEP levels were significantly higher in patients with PCOS than in the control group (140). A meta-analysis showed that non-obese PCOS patients had significantly higher circulating LEP levels than non-obese healthy women (141). In animal experiments, DHT-induced excess androgens in PCOS rats increased neuropeptide Y expression by down-regulating insulin and LEP signaling in the hypothalamus. This increases the rats’ food intake and promotes obesity (142). Higher LEP levels in women with PCOS may be related to a number of factors, among which insulin has been shown to induce more LEP secretion in white adipose tissue and increase circulating LEP levels (143, 144). LEP receptors are enriched in the hypothalamus (145), a region rich in neurons that control energy homeostasis and autonomic nervous function, and LEP binding to its receptors can increase the sympathetic nerve activity that innervates many organs (146). Microinjection of LEP into the hypothalamic arcuate nucleus and paraventricular nucleus of rats can induce sympathetic nerve excitation (147), and LEP may increase sympathetic nerve activity partly through local activation of melanocortin 3/4 receptor (148). SHU9119 blocking melanocortin 3/4 type receptors in the paraventricular nucleus of the hypothalamus reduced sympathetic nerve activity in LEP treated rats (149). Studies have shown that LEP interacts with sympathetic nerve fibers in adipose tissue and is involved in the lipolysis of white adipose tissue (150), and may be involved in more energy metabolism processes.

CHEM is mainly secreted by white adipose tissue and is involved in biological processes such as immune regulation, adipogenesis and energy metabolism (151). CHEM and its chemokine-like receptor 1 are richly expressed in the ovary and have an inhibitory effect on the production of ovarian steroid hormones (152). In human primary granulosa cells, CHEM treatment inhibited the expression of aromatase and cytochrome P450 in follicles, further impairs the secretion of E2 and progesterone (153). In DHT-induced PCOS rats, hyperandrogenemia increases the expression of chemerin and chemokine-like receptor 1 in the ovary and may therefore affect the immune microenvironment of the ovary, leading to local ovarian inflammation (154). A retrospective study reported increased CHEM expression in patients with PCOS, and serum CHEM concentrations can reflect the severity of polycystic changes (155). Metabolic factors such as obesity and IR affect CHEM expression in PCOS patients. Studies have shown that compared with healthy women, serum CHEM expression is higher in PCOS patients, and serum CHEM levels are higher in PCOS women with higher BMI than PCOS women with lower BMI (156, 157). CHEM, on the other hand, are known to be inhibitors of insulin signaling and glucose catabolism, weakening gene expression in cells involved in glucose and lipid homeostasis (158). Clinical studies have observed significant increases in chemerin levels in serum, subcutaneous, and omental adipose tissue in patients with PCOS, further increases after insulin infusion, and declines after metformin treatment (159). CHEM expressed in the hypothalamus and pituitary gland and have a potential role in controlling neuroendocrine events, and intraventricular injection of chemokine-9 increases plasma adrenaline levels and sympathetic nerve activity, which is mediated by the CMKLR1 receptor (160). Chemotactic proteins have been shown to alter sympathetic contractions to control blood pressure (161). This may indicate that the autonomic nerve exerts its influence on the body through its interaction with adipokines.

### 6.2 Participating in inflammatory pathways

Inflammation is one of the key factors affecting the pathology of PCOS. Studies have shown that compared with normal women, PCOS patients have significantly higher levels of serum and ovarian inflammatory markers (IL-2, IL-6, IL-18, IL-8, IFN-γ, TNF-α, etc.) and lower concentrations of anti-inflammatory cytokines (IL-10) (162, 163). The imbalance between pro-inflammatory and anti-inflammatory cytokines breaks down physiological homeostasis and generates inflammation, resulting in a microenvironment of low-degree chronic inflammation in PCOS patients, leading to the occurrence and development of PCOS (164). IR and hyperandrogenemia are considered to be the main causes of PCOS. Studies have shown that inflammation in tissues of PCOS patients interacts with impaired insulin metabolism (165). Local inflammatory environment can induce abnormal insulin signal transduction to cause IR, which is accompanied by hyperglycemia, and glucose can lead to increased levels of pro-inflammatory cytokines. This induces local and systemic proinflammatory states and impairs insulin signaling (166). High levels of androgens can activate the NF-κB inflammatory signaling pathway, resulting in the increase of CRP, TNF-α, IL-6 and other inflammatory factors, and induce the body’s inflammatory response. Inflammatory factors such as IL-6 can also activate the JAK/STAT3 signaling pathway, thereby inhibiting GLUT-4 secretion and preventing normal glucose metabolism (167). Chronic inflammation also affects follicular development, leading to ovarian dysfunction, and abnormally elevated levels of pro-inflammatory factors have been shown to inhibit the proliferation and differentiation of PCOS follicles, resulting in delayed follicular maturation (168).

In the inflammatory response, signals of the inflammatory state are communicated to the central nervous system, and the brain integrates the information and plays an important role in immune regulation, in which the autonomic nervous system plays an important role. Inflammatory signals are transmitted from the afferent fibers of vagus nerve to the nucleus tractus solitaris, and then activate the paraventricular nucleus of hypothalamus to secrete antidiuretic hormone and adrenocorticotropin releasing hormone, which activate the anterior pituitary gland and release adrenocorticotropin, corticotropin activates adrenocortical cells, which further release adrenocortical hormones. The final release of the most potent anti-inflammatory hormone, glucocorticoids, exerts long-lasting anti-inflammatory effects, namely the hypothalamic-pituitary-adrenalin axis (HPA) anti-inflammatory pathway (169, 170). Inflammation also activates the cholinergic anti-inflammatory pathway, where inflammatory signals are transmitted to the brain through the afferent vagus nerve and then integrated in the central part of the brain, and then these integrated immunoinflammatory signals are transmitted to the efferent vagus nucleus. Finally, it is activated at the efferent fibers of the vagus nerve, triggering the release of neurotransmitter ACh from external nerve endings. On the surface of immune cells (macrophages), ACh effectively inhibits the release of pro-inflammatory factors in macrophages by binding to the specific nicotinic Ach receptor, and weakens the inflammatory response, thus playing a role in protecting the immune system (171, 172). The splenic sympathetic anti-inflammatory pathway also plays an important role. After the inflammatory message is transmitted to the spleen, the vagus nerve in turn drives the splenic sympathetic nerve to release NE in the spleen (173), which activates β2 adrenergic receptors expressed by specific subsets of T cells that are capable of synthesizing and releasing Ach. Ach binds to α7 nicotinic receptors in macrophages, thereby inhibiting the release of pro-inflammatory cytokines (174). The regulation of inflammatory processes by ANS may be an important mechanism involved in the development of PCOS.

### 6.3 Affecting the digestive system

The autonomic nervous system is a key component of a two-way communication system between the central nervous system and the gut, where the gut microbiome, the gut, and the central nervous system form a microbiome-gut-brain axis that plays a crucial role in several aspects of physiology, including regulation of eating and appetite, glucose homeostasis, and intestinal motility (175, 176). ANS neurons are found in the celiac, superior and inferior mesenteric ganglia, which governs intestinal function. These neurons transmit information about intestinal homeostasis, including intestinal fluid exchange, pancreatic body secretion, mucosal barrier function, etc. to the CNS (177, 178). Gastrointestinal hormones cholecystokinin, glucagon-like peptide-1, and casein are secreted from intestinal mucosal cells in response to nutrient intake signals and can act on the afferent subgroup of the vagus nerve (179, 180). Stress caused by food intake can also stimulate the sensory neurons of the vagus nerve, through which information reaches the brain. Triggers the activation of outgoing vagus signals in the brain, which in turn are involved in the neural control of food intake (181). Glucagon-like peptide-1 receptor vagus afferent activation improves glucose tolerance and its inhibition increases blood glucose levels (182). There is two-way communication between the neuroendocrine system and the gut microbiome, with a network of specialized target cells/transducers in the gut wall acting as an interface between the microbiome and the host cavity. In response to external and physical demands, the brain regulates these specialized cells in this network through the branches of ANS. The microbiome maintains continuous two-way communication with this interface through multiple microbial signaling pathways that are regulated in response to perturbations in the microbiome or brain (183, 184). The disturbance of intestinal microbiota and gastrointestinal hormone secretion in PCOS patients can affect the gastrointestinal system and the central nervous system through the vagus nerve (185, 186), and its specific role needs to be further studied.

## 7 Clinical evidence of autonomic nerve regulation for PCOS

Given the role of the ANS in PCOS, regulating autonomic activity may be an effective approach to managing PCOS. In terms of surgical treatment, ovarian wedge resection can increase ovulation in PCOS patients, part of the reason may be that the procedure destroys the sympathetic nerve that innervates the ovaries (187). Clinical research has shown that two PCOS patients with concomitant refractory hypertension experienced reduced MSNA and whole-body NE overflow three months after bilateral renal neuroablation. Not only did their blood pressure decrease, but their insulin sensitivity also significantly improved, and one woman regained her menstrual cycle (188). Rational exercise is an effective method for treating PCOS. Clinical studies have demonstrated that after 16 weeks of aerobic exercise, obese PCOS patients can experience increased vagal nerve regulation (RMSSD, HF, HFnu, 2UV%), reduced sympathetic modulation (LF, LFnu), and lower resting heart rate and SBP (189). Other research has indicated that aerobic exercise training can lower body weight and insulin area under the curve in overweight and obese PCOS women, meanwhile levels of the inflammatory markers CRP and white blood cells were reduced, moreover, the improvement of the above metabolic and inflammatory indexes is correlated with the improvement of HRR after exercise (190). However, when PCOS women underwent HRV measurements after 4 months of anaerobic exercise, there were no significant changes in the parameters (191). Clinical research also suggests that aerobic exercise is more beneficial for PCOS patients compared to anaerobic exercise.

**Figure 1:**
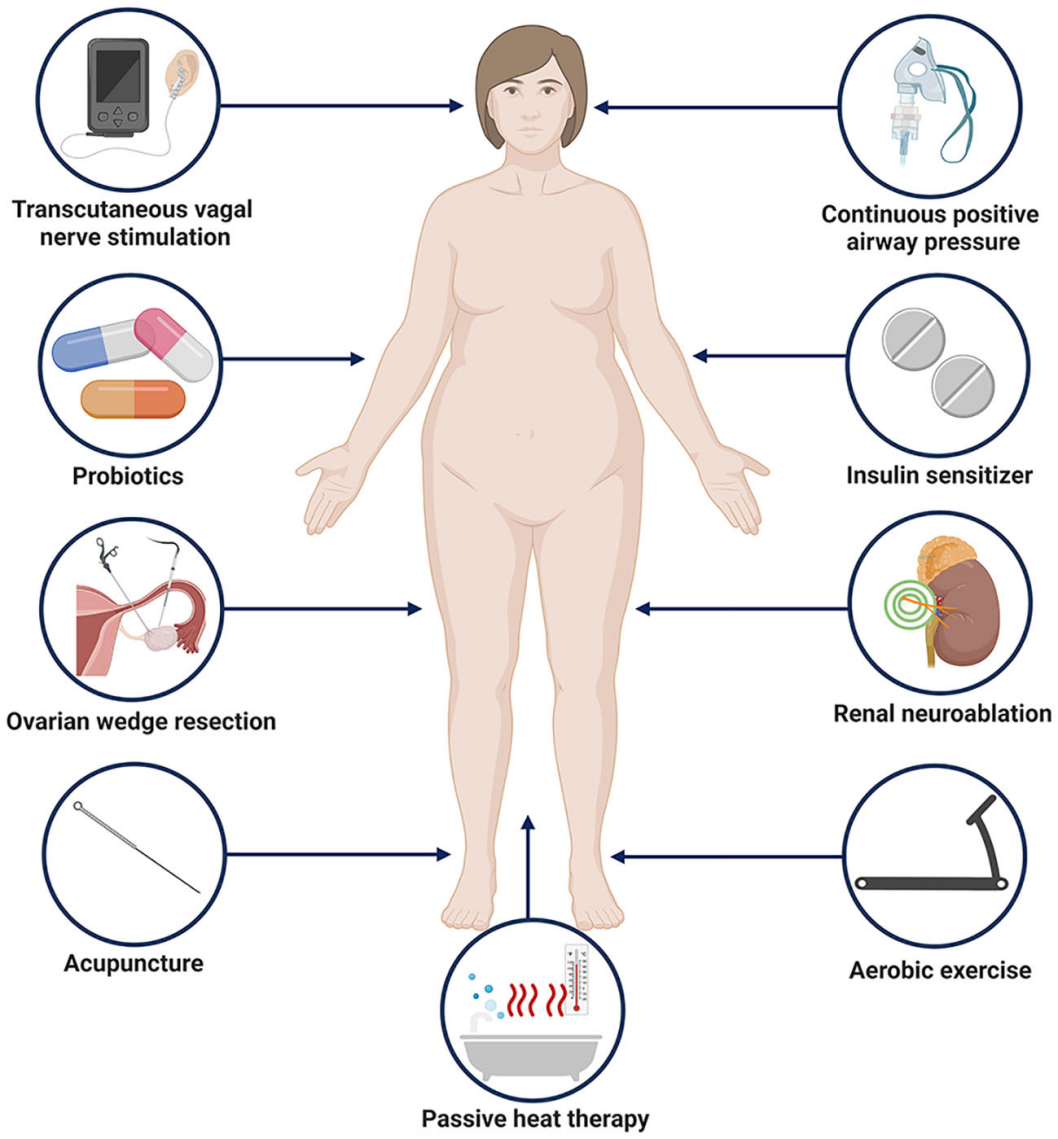
Potential approaches to PCOS intervention based on regulation of autonomic nerves. Created with BioRender.com.

OSA refers to the recurrent occurrence of apnea and/or hypopnea during sleep, resulting in chronic intermittent hypoxemia, hypercapnia, and disruptions in sleep architecture. As previously mentioned, PCOS women have a higher prevalence of OSA (192), the pathogenesis of OSA is associated with increased sympathetic nervous system activity (193, 194). Continuous positive airway pressure (CPAP) therapy is the most commonly used method to treat OSA (195). In young obese women with PCOS who received CPAP, subjects showed improved insulin sensitivity and reduced plasma 24-hour NE levels with no change in body weight, as found by HRV testing sympathetic output is reduced, and treatment transforms cardiac autonomic nerve activity to lower sympathetic tone and higher parasympathetic tone (196). Clinical studies have exposed subjects to repeated passive heat exposure through hot water baths, which improved flow-mediated vasodilation, protected vascular endothelium from ischemia-reperfusion-related injury, lowered total cholesterol levels, and fasting blood sugar. After treatment, subjects exhibited decreased MSNA and total T levels, with MSNA decreasing earlier than T, indicating that changes in sympathetic nervous system activity precede changes in androgen production. This provides support for the theory that sympathetic nervous system activity drives ovarian androgen production (197). Acupuncture may regulate sympathetic nervous system activity by stimulating ergot receptors and somatic afferents in muscles to modulate sympathetic activity. Compared to untreated control groups, low-frequency EA reduced MSNA burst frequency and free T in overweight PCOS women (198). Therefore, the ANS may offer new therapeutic targets for PCOS, but larger-scale and long-term studies are needed before applying these treatments in clinical practice. Based on our previous discussion of the mechanism, potential treatments include altered gut microbiota that normalizes signals to the autonomic nervous system, and probiotics are a good option (199). Transcutaneous auricular vagus nerve stimulation is a nerve regulation technique that can enhance the excitability of vagus nerve by stimulating the vagus nerve of the auricular appendicular cavity, which helps to restore the balance of autonomic nerve function (200). It is the most widely used and safest way of transcutaneous nerve stimulation. Nerve impulse is gradually transmitted to the brain region of the central nervous system through the vagus nerve along the dorsal raphe nucleus, parbrachial nucleus, hippocampus, prefrontal cortex, etc., thus achieving the regulatory effect on organs (201). This therapy has been used in the treatment of a variety of autonomic nervous disorders such as depression, obesity, hypertension and abnormal glucose tolerance, and has achieved good clinical efficacy (202, 203). With reduced vagus nerve activity in patients with PCOS, taVNS may be a potential treatment for PCOS (204). Since IR is a hallmark of PCOS and insulin may be involved in activation of the sympathetic nervous system, insulin sensitizers may be considered to have therapeutic benefits in reducing sympathetic output in PCOS. Studies have shown that metformin therapy can normalize cholinergic response in obese rats through M3 muscarinic acetylcholine receptors in pancreatic beta cells and improve ANS function in obese rats, suggesting that metformin may act on the ANS to treat PCOS (205). Intervention of PCOS based on adjustment of autonomic nerve were shown in **Figure 1**.

## 8 Discussion

The ANS plays a significant role in the development and progression of PCOS, affecting aspects such as follicular growth and development, steroid hormone secretion, and glucose and lipid metabolism regulation. Mechanisms include the activation of the central nervous system by afferent nerves, direct influences on ovarian function by regulating ovarian blood flow and hormone secretion, and involvement in the body’s inflammatory immune response. MSNA and HRV are commonly used methods in clinical practice to assess autonomic function in PCOS patients. PCOS women often exhibit enhanced sympathetic nervous system activity and reduced vagal nerve activity. Interventions based on regulating the ANS have already been explored for PCOS management. However, we contend that there remain several issues in this field that warrant further investigation. As previously mentioned, the ANS works in parallel with the HPOA, regulating follicular development and ovulation. The fluctuation of female hormones affects the remodeling of central neurons and the sensitivity of adrenergic receptors, thereby influencing autonomic nervous function. Furthermore, different sex hormones may play varied roles, but the specific mechanisms of action are not yet clear. Future research could explore the distinct mechanisms by which varying levels of sex hormones modulate autonomic nervous function in patients with PCOS and their potential targets. We also wish to highlight that studies based on rodent models have provided substantial evidence of the ANS’s involvement in the development and progression of PCOS. This inspires us to pursue more explorations in future clinical work, including the identification of more serological markers that reflect autonomic activity with high specificity, investigating targeted therapeutic approaches from the relatively independent perspective of the ANS, and conducting more randomized controlled trials to evaluate the clinical efficacy of autonomic-related treatments in PCOS patients. Moreover, research on the concept and implementation of vagus nerve modulation in treating PCOS is scant, representing a potentially promising area of study. However, it must be noted that as the ANS controls cardiopulmonary functions, many therapeutic approaches may carry the risk of cardiopulmonary side effects. Therefore, exploring ways to mitigate these risks is also of significant interest.

## Author contributions

YYu: Writing – original draft, Writing – review & editing. TC: Writing – original draft, Writing – review & editing. ZZ: Writing – original draft. FJ: Writing – original draft. YaL: Visualization, Writing – review & editing. YR: Visualization, Writing – review & editing. XL: Writing – review & editing. YL: Conceptualization, Visualization, Writing – review & editing.
